# Rickettsiosis in Southeast Asia: Summary for International Travellers during the COVID-19 Pandemic

**DOI:** 10.3390/tropicalmed7020018

**Published:** 2022-01-27

**Authors:** Thundon Ngamprasertchai, Borimas Hanboonkunupakarn, Watcharapong Piyaphanee

**Affiliations:** Department of Clinical Tropical Medicine, Faculty of Tropical Medicine, Mahidol University, Bangkok 10400, Thailand; thundon.ngm@mahidol.ac.th (T.N.); watcharapong.piy@mahidol.ac.th (W.P.)

**Keywords:** rickettsiosis, Southeast Asia, travellers, scrub typhus, murine typhus, coronavirus disease 2019, COVID-19

## Abstract

Rickettsiosis is an important cause of febrile illness among travellers visiting Southeast Asia (SEA). The true incidence of rickettsiosis is underestimated; however, murine typhus and scrub typhus are widely distributed across SEA. Among travellers visiting SEA, scrub typhus was mostly reported from Thailand, whereas murine typhus was frequently found in Indonesia. Although most cases are self-limited or present with mild symptoms, a few cases with severe clinical manifestations have been reported. Doxycycline remains the key treatment of rickettsiosis. Some travellers, such as backpackers, trekkers, or cave explorers, are at a higher risk for rickettsiosis than others. Therefore, in resource-limited conditions, empirical treatment should be considered in these travellers. The coronavirus disease 2019 (COVID-19) pandemic has contributed to difficulty in the diagnosis of rickettsiosis because of the clinical similarities between these diseases. In addition, physical distancing mandated by COVID-19 management guidelines limits accurate physical examination, resulting in misdiagnosis and delayed treatment of rickettsiosis. This review summarises the characteristics of murine typhus and scrub typhus, describes travel-associated rickettsiosis, and discusses the impact of the COVID-19 pandemic on rickettsiosis.

## 1. Introduction

Rickettsiosis ranks fourth among the identifiable aetiologies for febrile immigrants and returning travellers (mean 1.7% of febrile cases (0–7%)) from Africa, followed by those from the continent of Asia [1]. Southeast Asia (SEA) is a region of growing tourism where millions of travellers visit annually for both urban and ecotourism activities. Previous studies reported that rickettsiosis as a cause of systemic febrile illness in SEA was as uncommon as malaria or dengue fever [2]. However, the true epidemiology of rickettsiosis has been underestimated; for example, about 67% of the patients were misdiagnosed with scrub typhus [3]. This underestimation is attributed to under-recognition, under-testing of possible cases and the lack of standard laboratory testing. Moreover, an ill traveller who is febrile may present with non-specific constitutional symptoms. In addition, eschar may manifest in specific types of rickettsioses [3]. Physicians should have a high index of suspicion for international travellers who return home with fever after travelling from SEA [4].

Rickettsiosis is a zoonotic bacterial infection transmitted to humans by several arthropods, including lice, fleas, ticks, and mites. Rickettsiosis is caused by obligate intracellular Gram-negative rod bacteria in the family *Rickettsiaceae* and can be classified into three groups: (1) typhus group, which contains two species: endemic murine typhus or *Rickettsia typhi* and epidemic typhus or *Rickettsia prowazekii*; (2) spotted fever group; and (3) scrub typhus group of the genus *Orientia tsutsugamushi* [5]. Spotted fever group rickettsiosis (SFGR) comprises many species. *R. africae*, the causal agent of African tick bite fever, is a common rickettsiosis found in travellers [6]. It commonly occurs in clusters among safari tourists travelling from Africa [7]. In Southeast Asia, there are several SFGRs that have been reported to cause human infection such as *R. honei*, *R. conorii* subsp. *indica*, *R. helvetica*, *R. japonica*, and *R. felis*. *O. tsutsugamushi* was originally known as *Rickettsia tsutsugamushi*. It is the only member recently separated from genus *Rickettsia* due to genotypic and phenotypic differences. *R. typhi*, *O. tsutsugamushi*, and some SFGRs, including *R. honei*, are important rickettsial species in SEA. However, both *R. typhi* and *O. tsutsugamushi* play a major role in rickettsiosis among SEA residents and travellers returning from SEA [8,9]. Although the mortality rate for treated cases is low, some reports have shown high mortality in untreated patients. Mortality among murine typhus cases not treated with antibiotics ranges from 0.4% to 4% [10,11], whereas the median mortality rate is 6% for untreated scrub typhus cases [12]. Therefore, prompt diagnosis and appropriate antibiotic use are the main factors that contribute to survival. This narrative review aims to enhance the recognition and understanding of rickettsiosis, particularly SFGR, murine typhus, and scrub typhus, which are widely reported in SEA. This review also discussed the challenges and summarises the published case reports of rickettsiosis among travellers during the coronavirus disease 2019 (COVID-19) pandemic.

## 2. Spotted Fever Group Rickettsiosis (SFGR)

The SFGRs are transmitted to humans from rodents, dogs, and wild animals, mostly by ticks and occasionally by lice. This review focuses on common species which are specific to SEA. *R. honei* or Thai tick typhus is one of the SFGRs endemic in Thailand [13]. Most reported cases were from rural areas in SEA. Symptoms are usually mild and an eschar might be seen. It was used as a reference species in serologic diagnosis tests in SEA [14]. *R. felis* is transmitted to humans by cat fleas. The clinical syndrome is similar to murine typhus [15]. *R. helvetica* has been reported along the Thailand–Myanmar border [16]. The symptoms are mild and the rash is absent [16].

## 3. Murine Typhus

Endemic murine typhus caused by *R. typhi* is widely distributed across tropical and subtropical regions. Both the local population and travellers are affected by this disease. *R. typhi* is transmitted to human by fleas. Rats and other rodents act as reservoirs. The classical transmission is rat–flea–rat. Humans are accidental hosts. In addition to these rats, other commensal mammals such as cats, shrews, or opossums are potential sources of human infection [17]. Travellers should avoid unnecessary animal contact. Several factors contributing to the transmission of murine typhus include overcrowding, poor sanitation, and urbanisation that expose humans to rodent populations. Consequently, the populations at risk are individuals in port cities or coastal regions with rodents [5], those living in refugee camps [18], and city dwellers or immigrant workers living in poor and unhygienic conditions [14]. Murine typhus presents with benign or self-limiting symptoms within 7 days of infection [14]. Fever and non-specific symptoms, such as headache, rash, arthralgia, or gastrointestinal symptoms, are the presenting manifestations [10,19]. Doxycycline is the mainstay for treatment, and its primary benefit is a shorter fever clearance time [20]. Untreated patients have a longer duration of fever with a higher rate of overall complications (26%) [21], such as meningoencephalitis, pneumonia, acute kidney injury, and septic shock.

## 4. Scrub Typhus

**Figure 1:**
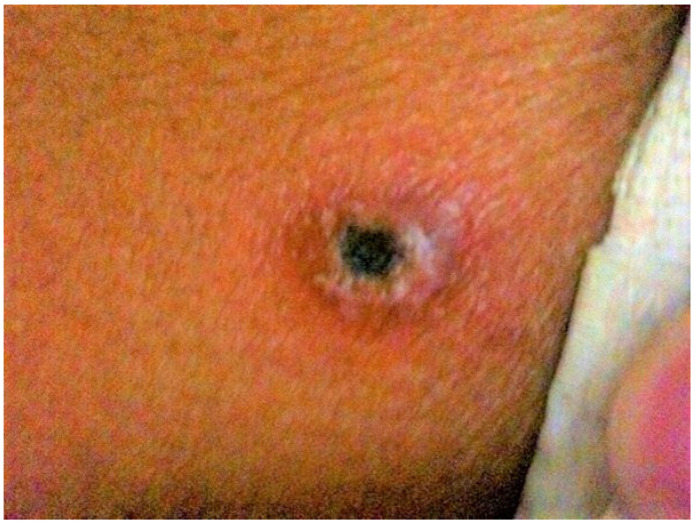
Eschar on the back of a patient with scrub typhus. Unidentified patient photo from Thundon Ngamprasertchai, MD.

Unlike murine typhus, scrub typhus is an infection transmitted by mites (also termed chiggers). Scrub typhus is a serious infection: 1.4% of patients die if treated, and mortality rate can reach 24% with multi-organ failure [12]. *O. tsutsugamushi* is acquired from the bite of an infected mite, which are commonly found on rodents. Rural populations are mainly affected, but infections in urban areas are also increasing. The incidence of infection varies seasonally, with an uptrend towards the end of the rainy season (May–August) [22]. Although most scrub typhus cases are mild, severe clinical manifestations are more likely to occur as the inflammatory response increases. Clinical presentation includes acute-onset fever, myalgia, rash, lymphadenopathy, and conjunctival injection. Approximately 50% of cases manifest with an eschar, which is possibly diagnostic (Figure 1). Eschars typically present on the axilla or groin. The absence of eschars might result in delayed diagnosis, complications, and high mortality. Doxycycline or chloramphenicol are the treatment options for scrub typhus. Rifampicin monotherapy and combination therapy with doxycycline have also been reported [23].

## 5. Travel-Associated Rickettsiosis 

More than 450 travel-associated cases of rickettsiosis have been reported worldwide [5]. *R. typhi* and *O. tsutsugamushi* infections are the vast majority of rickettsiosis aetiologies in travellers who visited SEA [8,9]. Travellers frequently have symptoms before or within a few days of return from endemic countries because the incubation period for rickettsiosis is 6–20 days [14]. Most symptoms among travellers are mild; however, severe complications have also been reported, particularly in Thailand [23,24].

The true incidence of rickettsiosis in SEA is unknown [25,26]. A study from Thailand reported that the incidence of scrub typhus as the cause of febrile illness in the country was 5.6%, which was even higher in the northern region [27]. In contrast, murine typhus was more prevalent than scrub typhus (5% vs. 1%) among 397 patients with acute undifferentiated fever at the Bangkok Hospital for Tropical Diseases, Thailand [28]. A recent study from Vietnam reported that among 1127 non-malarial febrile patients, scrub typhus was the aetiology of fever in 33 cases (2.9%) [29]. However, the proportion of scrub typhus was possibly as high as 36% in a study of an agricultural area of Sabah, Malaysia [30].

Not only the local population but also those travelling to SEA are at risk of contracting rickettsiosis [31]. Nevertheless, all studies assessed only returned travellers; therefore, it is difficult to determine the true incidence of rickettsiosis because of the lack of denominator data. In addition, the reported cases among returned travellers are more likely to be underestimated because the incubation period of rickettsiosis is short. We anticipate that some of those travelling to SEA may have contracted scrub typhus and developed symptoms during their trip. Therefore, the true number of rickettsiosis cases reported among returned travellers could be underestimated. Hence, robust epidemiological studies among travellers are needed.

A noteworthy study was published by the GeoSentinel Surveillance Network in 2009 [9]. Their findings for 47,915 travellers assessed from 1996–2008 showed that 0.6% (280) had rickettsiosis, representing a morbidity rate of 1.5%. Among the 280 travellers, the most common infectious group was the spotted fever group (82.5%, 231), which is frequently found in travellers returning from Sub-Saharan Africa. A few cases of scrub typhus were also reported, of which >50% were from SEA. Although the incidence of scrub typhus among travellers was low and the disease was self-limited, a few reported cases, particularly from SEA, were fatal.

A retrospective study assessed the laboratory data of Swedish travellers from 1997–2001 and showed that 14 of 77 total cases of rickettsiosis were caused by the typhus group [32]. However, the results of previous epidemiological studies of rickettsiosis should be interpreted with caution considering the variability in diagnostic tests.

We anticipate that the risk of rickettsiosis in SEA will remain high because of the reservoirs and vectors that exist throughout the region. Travellers who engage in risky activities, such as trekking, camping, or rafting in rural areas, are likely to be exposed to the zoonotic life cycle of pathogens; therefore, backpackers and ecotourism travellers are at risk. Several places have been reported as most suitable for disease transmission, for example, refugee camps [18], unhygienic residences of immigrants [5], and agricultural areas [33]. In addition, logging, land clearing, road building, and military operations are also considered to be high-risk activities for infection. Nevertheless, the disease may also occur in high socioeconomic status settings; for example, travellers staying in a luxurious hotel in Bali were reported to contract murine typhus [34]. During World War II and the Vietnam War, scrub typhus was widespread among troops [35].

Travel medicine physicians or physicians living in non-endemic areas should make a presumptive diagnosis based on patient history and exposure risk because confirming the diagnosis of rickettsiosis is difficult. Indirect immunofluorescence assay (IFA) is a gold standard serologic diagnosis for rickettsiosis [10,36]. However, there are several limitations. For instance, paired serum samples are needed for a more than 4-fold increase between acute and convalescent phase serology titers [14]. Next, serum may cross-react with other bacteria such as *Anaplasma* spp. or *Ehrlichia* spp. [37]. Lastly, prior antibiotic effects may reduce antibody response [36]. Molecular techniques such as nucleic acid amplification tests (NAATs) or the PCR method may enable diagnosis rather than serology but they are not widely available and are restricted to research fields [36]. Therefore, a rapid point-of-care test is needed for clinicians working at travel clinics. In addition, an empirical treatment of doxycycline would benefit travellers by preventing further complications.

Travellers can also take steps to minimise their chance of infection. They should avoid risky activities and places during their stay in an endemic area. They should not touch rodents or local dogs and cats. Bush vegetation should not be entered. General preventive measures for arthropod-borne diseases should also be undertaken. For example, the use of protective clothing impregnated with permethrin and topical repellents is recommended. Daily checking for mites and ticks and appropriate removal are also emphasised. Chemoprophylaxis for rickettsiosis with 200 mg doses of doxycycline has been established for scrub typhus [38,39]. This approach should be the choice for high-risk travellers, such as backpackers, trekkers, and cave explorers, especially in areas where access to a medical facility is limited. Currently, vaccination against rickettsiosis has been investigated in an experimental animal model [40]. Unfortunately, to date, there is no promising human vaccine in the drug pipeline.

## 6. Reported Cases of Scrub Typhus and Murine Typhus in Travellers from SEA

All cases of travel-acquired scrub and murine typhus in SEA were searched for in MEDLINE and SCOPUS from 2000 and 2021. Case reports and case series were selected with an English language restriction. The followings search terms were used: ((travelers) OR (travel[MeSH Terms])) AND (((murine typhus[MeSH Terms]) OR (scrub typhus[MeSH Terms])) AND (infection, rickettsia[MeSH Terms])). We excluded the studies without diagnosis confirmation and the studies of travellers who acquired the disease outside SEA. The search was performed on 25 October 2021.

**Figure 2:**
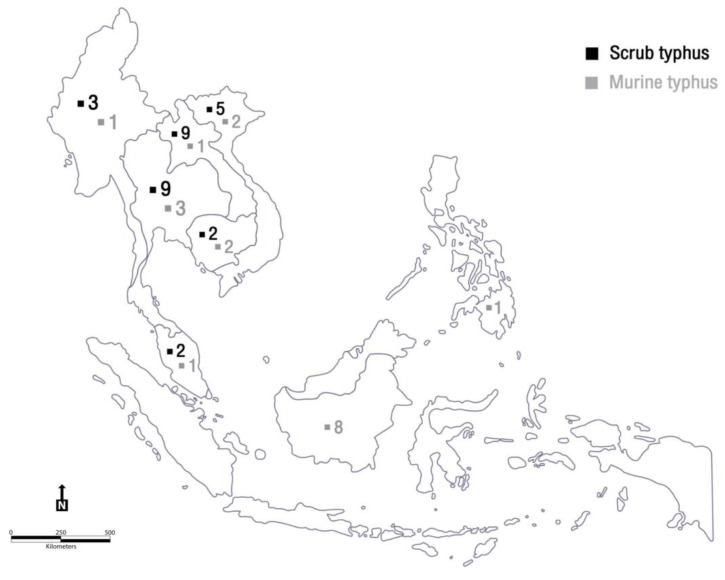
Demonstration of reported scrub typhus and murine typhus cases in Southeast Asia. Figure by Mrs. Siwaporn Panphoowong (Adobe Illustrator).

Demographic and epidemiologic features of the international travellers diagnosed with scrub typhus and murine typhus are presented in Table 1 and Table 2, respectively. Disease distributions are shown in a regional map in Figure 2. Most reports of affected travellers were of young adults from Europe. Scrub typhus cases were mostly reported from Thailand and Laos, whereas most murine typhus cases were reported from Indonesia. Sporadic cases were frequently documented. This was unlike African tick bite fever, which is caused by *Rickettsia africae* and usually occurs in clusters or outbreaks among travellers who engaged in risky activities while on safari. Ecotourism activities such as trekking, hiking, or walking through bushes were common among sick travellers; therefore, pre-travel consultation should emphasise prevention measures. Nevertheless, no cases of mortality were noted among the travellers. This was attributed to physicians having a high index of suspicion for patients with a history of travelling to SEA. Empiric treatment with doxycycline was promptly administered.

## 7. Impact of the COVID-19 Pandemic on Rickettsiosis

Since the COVID-19 pandemic began, several countries have used a lockdown policy to limit the spread of the disease. Both local and international travel restrictions have been widely imposed worldwide. Prioritisation of travel, particularly for emergencies or humanitarian purposes, is recommended. According to the World Tourism Organization, global international tourist arrivals in January 2021 showed an 87% reduction compared with 2020 and a 96% reduction for the Asia and Pacific regions [60]. However, these travel restrictions may have only moderately reduced COVID-19 transmission unless combined with other interventions [61]. Some additional beneficial measures include physical distancing, proper mask wearing, frequent hand washing, and avoiding crowded areas. Some of these practices can also reduce the chances of infection caused by the pathogens of various diseases in both local people and travellers.

A recent study from Taiwan revealed that the summer incidence of scrub typhus in 2021, when national lockdown measures and travel restrictions were implemented, was reduced to <50% of the average summer incidence over the past 5 years [31]. We expect that international travellers will show a similar trend. Most patients with COVID-19 have respiratory symptoms because the virus primarily affects the respiratory system. The respiratory manifestations of COVID-19 can range from cough to severe pneumonia. Cough was also noted in approximately 30% of patients with murine typhus and 50% of those with scrub typhus [22,62]. However, cough may be less common among children [63]. While most COVID-19 variants can present as an acute febrile illness or cough, which is similar to rickettsiosis, the recent emerging omicron variant frequently presents similarly to the common cold. Fever and cough are less likely than with either the delta or alpha variant [64]. Hence, clinicians should consider other clinical manifestations to differentiate between the two diseases.

In addition to febrile illness, skin manifestations are common in COVID-19 as well as murine typhus and scrub typhus. The incidence of rashes in cases of murine and scrub typhus varies from <10% [22] to >50% [14], and rash typically occurs a few days after the onset of fever. The characteristics of the rash, such as vasculopathic lesions in COVID-19, can resemble those of an eschar in scrub typhus and other eschar-causing rickettsioses [65].

The rising concern over the COVID-19 pandemic and the overlapping signs and symptoms between COVID-19 and rickettsiosis can complicate the diagnosis and management of patients with these diseases. Recently, Patel et al. reported the delayed diagnosis of a patient with murine typhus who presented with a 4-day history of fever, headache, and myalgia. Three tests for severe acute respiratory syndrome coronavirus 2 (SARS-CoV-2) were performed, all of which showed negative results. Subsequently, on day 14 of the illness, the diagnosis of murine typhus was made [66]. In another study, Ihara et al. reported a case of scrub typhus that was primarily diagnosed as COVID-19 [67]. The patient presented with a 7-day history of fever, generalised maculopapular rash, headache, and cough. When polymerase chain reaction testing for SARS-CoV-2 showed negative results, additional history taking and physical examination were performed, which revealed potential exposure to risk factors and an eschar. In addition, co-infection with COVID-19 and rickettsiosis is possible, as reported in India [68]. Interestingly, COVID-19 was the first differential diagnosis in all the reported cases, and the eschars were detected after testing for COVID-19. These case presentations emphasise the importance of increasing disease awareness, thorough history taking and physical examination, all of which can be challenging during the pandemic, when physicians may have a bias in diagnosis and may be experiencing a significant burden of workload.

The lack of appropriate history taking and physical examinations contributes to delayed diagnosis and management. A study of the effects of the COVID-19 pandemic on medical services in Taiwan revealed that physicians tended to be more cautious when examining patients, preferred to keep their distance and avoided prolonged contact. In addition, online medical consultations may be primarily used to reduce physical contact with patients [69]. These factors lower the possibility of discovering an eschar because it is painless and usually present in non-exposed areas.

Finally, the COVID-19 pandemic may reduce a traveller’s chance of contracting rickettsiosis in SEA due to lockdown measures and travel restrictions. However, COVID-19 may cause difficulty or delay in diagnosis and management if physicians are not aware of the potential for a rickettsial infection. Prompt treatment is recommended in cases of suspected rickettsial infection to prevent further complications.

## 8. Conclusions

Scrub typhus and murine typhus are the rickettsial diseases frequently reported among travellers from SEA. Pre-travel consultation is crucial for travellers, particularly for ecotourism. Both scrub typhus and murine typhus should be considered in travellers returning from SEA who present with a fever. The role of doxycycline is critical in treatment and prevention strategies. Empiric treatment with doxycycline should be promptly given. The COVID-19 pandemic may result in misdiagnosis or delayed treatment; therefore, physicians should be aware of the possibility of rickettsiosis when evaluating sick travellers.

## Figures and Tables

**Table 1:** Demographic and epidemiologic features of international travellers diagnosed with scrub typhus in Southeast Asia, 2000–2021.

Year	Age and Sex	Visited Countries	Citizenship	Risky Activities	Outcome
2000 [24]	32 F	Myanmar and Thailand	French	NA	Fully recovered
2004 [41]	49 M	Vietnam, Thailand and Myanmar	German	NA	Improvement
2006 [42]	40 F	Thailand	Swedish	Jungle trip in Chiang Mai	Discharged
	67 F	Laos	Swedish	Hiking along Mekong River	Improvement
2012 [43]	15 M	Thailand	German	Vacation on Koh Samui	Discharged
2013 [44]	31 F	Cambodia	French	Jungle trip	Improvement
2013 [45]	27 F	Laos	Dutch	Basic travelling through SEA	Recovered
2015 [46]	50 F	Laos	French	Jungle trip	Fever resolved
2017 [47]	23 F	Cambodia and Vietnam	Belgian	Hiking through high grass, bushes, and paddy fields	NA
	23 F	Thailand, Laos, and Vietnam	Belgian	Hiking through woods and caves and boat trip on the Mekong River	Fever resolved
2020 [48]	33 M 42 M	Malaysia and Borneo Thailand	Dutch Dutch	NA	NA
2020 [49]	55 M 50 F 34 M 35 M 27 F 51 M 61 M 19 M	Laos Laos and Thailand Vietnam Thailand and Malaysia Thailand Thailand Myanmar Vietnam	Data retrieved from German Reference Center for Tropical Medicine	NA	All resolved
2021 [50]	55 M 38 F 35 M	Laos Laos Laos	Italian Italian Italian	12-day hiking and camping trip to the forest of Northern Laos	Recovered

F = Female, M = Male, NA = Not Applicable.

**Table 2:** Demographic and epidemiologic features of international travellers diagnosed with murine typhus in Southeast Asia, 2000–2021.

Year	Age and Sex	Visited Countries	Citizenship	Risky Activities	Outcome
2001 [51]	37 M	Thailand	German	NA	Recovered
2006 [52]	54 M	Vietnam	Japanese	NA	NA
2010 [53]	23 M	Indonesia	Japanese	Surfing and stayed at guesthouses and local friends’ residences in Kuta and Madewi (Bali)	Improvement
	23 M	Indonesia	Japanese	NA	Improvement
2011 [54]	29 M	Indonesia	NA	Visiting both urban and rural areas in Bali and Lombok; seeing rat in his accommodation; and multiple insect bites but no tick bites	Improvement
2012 [55]	Retrospective 12 cases	Indonesia, Philippines, Thailand, Cambodia, Vietnam, Myanmar, or Laos	French	Tourism, business, and visiting friends and relatives	Recovered
2013 [56]	53 F	Indonesia	NA	Close contact with animals in safari park and multiple insect bites in Bali	Complete recovery
	59 F	Indonesia	NA	NA	Slow recovery
2013 [57]	56 M	Thailand	Japanese	Working as a Japanese language teacher	Improvement
2015 [58]	43 M	Bali and Indonesia	Japanese	NA	Improvement
2017 [47]	37 F	Indonesia and Malaysia	Belgian	A 24-day adventurous travel staying at local accommodation for several nights	Improvement
2018 [59] (only *R. typhi* confirmation)	57 M	Cambodia	German	NA	NA

F = Female, M = Male, NA = Not Applicable.

## Data Availability

All data are included in this manuscript. There are no other data involved in this study.
